# Towards systematic nomenclature for cell-free DNA

**DOI:** 10.1007/s00439-020-02227-2

**Published:** 2020-10-29

**Authors:** Abel J. Bronkhorst, Vida Ungerer, Frank Diehl, Philippe Anker, Yuval Dor, Michael Fleischhacker, Peter B. Gahan, Lisa Hui, Stefan Holdenrieder, Alain R. Thierry

**Affiliations:** 1 Institute for Laboratory Medicine, German Heart Centre, Technical University Munich, Lazarettstraße 36, 80636 Munich, Germany; 2 Thrive Earlier Detection Corp., Cambridge, MA USA; 3 IRCM, Institute of Research in Oncology of Montpellier, Montpellier, France; 4 INSERM, U1194, Montpellier, France; 5 University of Montpellier, Montpellier, France; 6 Department of Developmental Biology and Cancer Research, The Hebrew University-Hadassah Medical School, 91120 Jerusalem, Israel; 7 DRK Kliniken Berlin Mitte, Klinik für Innere Medizin, Pneumologie und Schlafmedizin, Drontheimer Str. 39-40, 13359 Berlin, Germany; 8 Fondazione “Enrico Puccinelli” Onlus, 06126 Perugia, Italy; 9 Reproductive Epidemiology Group, Murdoch Children’s Research Institute, Parkville, VIC Australia; 10 Department of Obstetrics and Gynaecology, University of Melbourne, Parkville, VIC Australia; 11 Department of Perinatal Medicine, Mercy Hospital for Women, Heidelberg, VIC Australia; 12 Department of Obstetrics and Gynaecology, The Northern Hospital, Epping, VIC Australia; 13 ICM, Regional Institute of Cancer of Montpellier, Montpellier, France

## Abstract

Cell-free DNA (cfDNA) has become widely recognized as a promising candidate biomarker for minimally invasive characterization of various genomic disorders and other clinical scenarios. However, among the obstacles that currently challenge the general progression of the research field, there remains an unmet need for unambiguous universal cfDNA nomenclature. To address this shortcoming, we classify in this report the different types of cfDNA molecules that occur in the human body based on its origin, genetic traits, and locality. We proceed by assigning existing terms to each of these cfDNA subtypes, while proposing new terms and abbreviations where clarity is lacking and more precise stratification would be beneficial. We then suggest the proper usage of these terms within different contexts and scenarios, focusing mainly on the nomenclature as it relates to the domains of oncology, prenatal testing, and post-transplant surgery surveillance. We hope that these recommendations will serve as useful considerations towards the establishment of universal cfDNA nomenclature in the future. In addition, it is conceivable that many of these recommendations can be transposed to cell-free RNA nomenclature by simply exchanging “DNA” with “RNA” in each acronym/abbreviation. Similarly, when describing DNA and RNA collectively, the suffix can be replaced with “NAs” to indicate nucleic acids.

## Introduction

The presence of cell-free DNA (cfDNA) in human blood was discovered in the 1940s (Mandel 1948), but only in the last 2 decades have researchers started to uncover the immense potential of cfDNA as a minimally invasive source of diverse biological and pathological information. Although cfDNA research is still a young field of inquiry, it is becoming clear that the utility of cfDNA spans multiple domains of both basic research and clinical diagnostics (Akirav et al. 2011; Amicucci et al. 2000; Atamaniuk et al. 2004; De Vlaminck et al. 2014; Diaz and Bardelli 2014; Fleischhacker and Schmidt 2007; Hui 2019; Lo et al. 1998; Stroun et al. 1989; Thierry et al. 2016; Wan et al. 2016; Zandvakili and Lazaridis 2019; Zemmour et al. 2018). In addition, cfDNA analysis represents a point of major advancement in the application of next-generation molecular techniques and bioinformatics towards minimally invasive patient-centered diagnosis, prognosis, prediction, and monitoring (Tuaeva et al. 2019).

While initially discovered in blood, cfDNA molecules have now been detected in all human body fluid types. The composition of the cfDNA population in any body fluid depends on several factors, such as the location of the fluid, the relative contribution of different organs and cell types, the mechanisms by which cfDNA is generated and released, the conditions surrounding its movement from immediate extracellular space into the specific body fluid, its interaction with other extracellular components, and its stability/half-life in the fluid. Concurrently, all these factors are modulated by various, often interconnected, biological and physiological factors, many of which are liable to notable intra- and interindividual variation (for comprehensive reviews, refer to Aucamp et al. 2018, Thierry et al. 2016, and Ungerer et al. 2020). As a result, the cfDNA profile in any body fluid is generally highly complex, often consisting of DNA fragments from diverse origins, including multiple organs, different cell types, and non-human endogenous and exogenous sources (e.g., microbial and viral DNA). Furthermore, it is now well understood that cfDNA molecules conserve, on a primary level, the unique genetic and epigenetic codes that characterize its originating source. In addition to the classical epigenetic marks (DNA methylation and histone modifications), cfDNA from different sources often carry unique information in the form of secondary structural features, such as protein-complexes, extracellular vesicle associations, and variable fragment size, end-point motifs, and nucleosome density (reviewed in Bronkhorst et al. 2019b). Despite these differences, cfDNA of different origins often display overlapping features. To list some examples, clonal hematopoiesis-derived cfDNA often exhibit cancer-associated mutations identical to circulating tumor DNA (ctDNA) (Gormally et al. 2006; Hu et al. 2018); ctDNA and wild-type DNA derived from different cell types often exhibit similar DNA methylation patterns and histone modifications; and ctDNA and cell-free fetal DNA (cffDNA) exhibit similar fragment sizes (Chan et al. 2004; Fan et al. 2010; Jiang et al. 2015; Mouliere et al. 2011; Sun et al. 2018; Sanchez et al. 2018).

Therefore, the characteristics of the cfDNA profile in a typical biospecimen source change dynamically, are highly heterogeneous, but also demonstrate overlapping physico-chemical features despite originating from disparate sources and processes. Historically, this inherently complex cfDNA mixture in most biospecimen types has not only complicated the analytical differentiation between cfDNA molecules of different origins, but also hampered the establishment of a proper nomenclature.

Due to the persistent lack of widespread consensus on nomenclature, the literature is flooded with numerous self-devised terms that describe different types of cfDNA. As summarized in Table 1, it is clear that (i) several variations of terms and abbreviations are often used to describe the exact same cfDNA type, (ii) the same terms are often used to describe cfDNA types that differ structurally and biologically, and (iii) incompatible terms are often used to describe the same cfDNA types. Although there is no direct evidence that inconsistent use of nomenclature presents a major obstacle to the development of clinically meaningful cfDNA tests, it does complicate interstudy comparisons and is a source of biased reading and referencing of literature. In addition, unclear and overlapping definitions often make it difficult for patients and genetic counsellors, for example, to understand the terminology. These drawbacks emphasize the need for unambiguous nomenclature (Otandault et al. 2019). As the cfDNA research field is currently in a strong growth phase with a rapid influx of new researchers, publications, as well as companies and associated stakeholders, it is an opportune time to address cfDNA nomenclature.

**Table 1:** List of the highly varied cell-free DNA (cfDNA) terms and their corresponding abbreviations or acronyms identified through exhaustive screening of the literature

Category	Term	Abbreviations found in the literature
Total DNA	Blood plasma DNA	N/A
	Cell-free circulating DNA	cfcDNA, fcDNA
	Cell-free DNA	cfDNA, cf-DNA, (cf) DNA, CFDNA
	Circulating cell-free DNA	cfDNA, ccfDNA, ccf DNA
	Circulating DNA	cirDNA, circDNA, C-DNA
	Circulating extracellular DNA	exDNA, ecDNA
	Circulating free DNA	cfDNA
	Extracellular DNA	exDNA
	Extracellular occurring DNA	eoDNA
	Free circulating DNA	fcDNA
	Free DNA	fDNA
	Plasma DNA	N/A
Total DNA and RNA	Cell-free chromatin	cfCh
	Cell-free nucleic acids	cf-NAs
	Circulating cell-free nucleic acids	cf-NAs, ccfNAs
	Circulating nucleic acids	cirNAs, cir-NA, CNAs
	Circulating nucleic acids in plasma and serum CNAPS	CNAPS
	Extracellular nucleic acids	exNA
Mitochondrial DNA	Cell-free mitochondrial DNA	cfmDNA, CFmDNA, cf-mtDNA, cf-mt-DNA
	Mitochondrial cell-free DNA	McfDNA
	Plasma cell-free mitochondrial tumor DNA	N/A
	Plasma tumor mitochondrial DNA	tmtDNA
	Whole blood mitochondrial DNA	wb-mtDNA
Nuclear DNA	Cell-free nuclear DNA	CFnDNA, cf-nDNA
	Circulating, cell-free nuclear DNA	ccf-nDNA
	Nuclear cell-free DNA	NcfDNA
Specific source	Cell-free seminal DNA	cfsDNA
	Cell-surface bound extracellular DNA	csbDNA
	Cerebrospinal fluid tumor DNA	CSF-tDNA
	Urinary cell-free DNA	ucfDNA
Oncology	Cell-free circulating tumor DNA	ctDNA, cfTDNA
	Cell-free tumor DNA	ctDNA, cftDNA
	Cell-free tumor-derived DNA	tDNA
	Circulating free tumor DNA	cftDNA
	Circulating tumor DNA	ctDNA
	Tumor DNA	N/A
	Tumor-derived circulating DNA	N/A
	Tumor-derived DNA	N/A
Prenatal testing	Cell-free fetal DNA	cffDNA, cff-DNA, fDNA, f-DNA
	Cell-free maternal DNA	cfmDNA
	Cell-free total DNA	cftDNA
	Circulating cell-free fetal DNA	cffDNA
	Circulating fetal DNA	cf-DNA, fDNA
	Fetal cell-free DNA	fDNA
	Fetal DNA	f-DNA, fDNA
	Free circulating fetal DNA	cfDNA
Post-transplant surgery surveillance	Cell-free donor-derived DNA	cfdDNA
	Donor-derived cfDNA	dcfDNA, ddcfDNA, dd-cfDNA
	Donor-specific cfDNA	cfdDNA
	Graft-derived cell-free DNA	GcfDNA

Indeed, a survey from the attendants of the 10th international CNAPS (Circulating Nucleic Acids in Plasma and Serum) meeting indicated that, despite different viewpoints on some aspects of the nomenclature, researchers in the cfDNA field recognize that there are several inconsistencies in cfDNA terminology and that there is a need for a unification of nomenclature (Otandault et al. 2019). Working towards such a unification, most of the Scientific Committee members of the 10th CNAPS meeting have cooperated to produce this report in which we have assigned what we consider to be suitable terms and abbreviations (both existing and new) to the different cfDNA types as it relates to different biological compartments and DNA origins, focusing on three highly investigated diagnostic areas: oncology, prenatal testing, and post-transplant surgery surveillance. As such, this proposal takes the shape of an exhaustive stratification of cfDNA subtypes. However, bearing in mind that such precise stratification of cfDNA subtypes in most clinical biospecimens would be unnecessary or impractical, the main goal of this proposal is to provide an expanded selection of nomenclature that could serve as a useful reference in specific scenarios for both basic researchers and clinicians. In line with this, it is important that the nomenclature suggested here should not be regarded as a consensus view of the broad cfDNA research community, but rather be scrutinized as a free-standing proposal representing the views of the authors. The secondary goal of this work is simply to raise awareness on the importance of proper nomenclature.

**Fig. 1:**
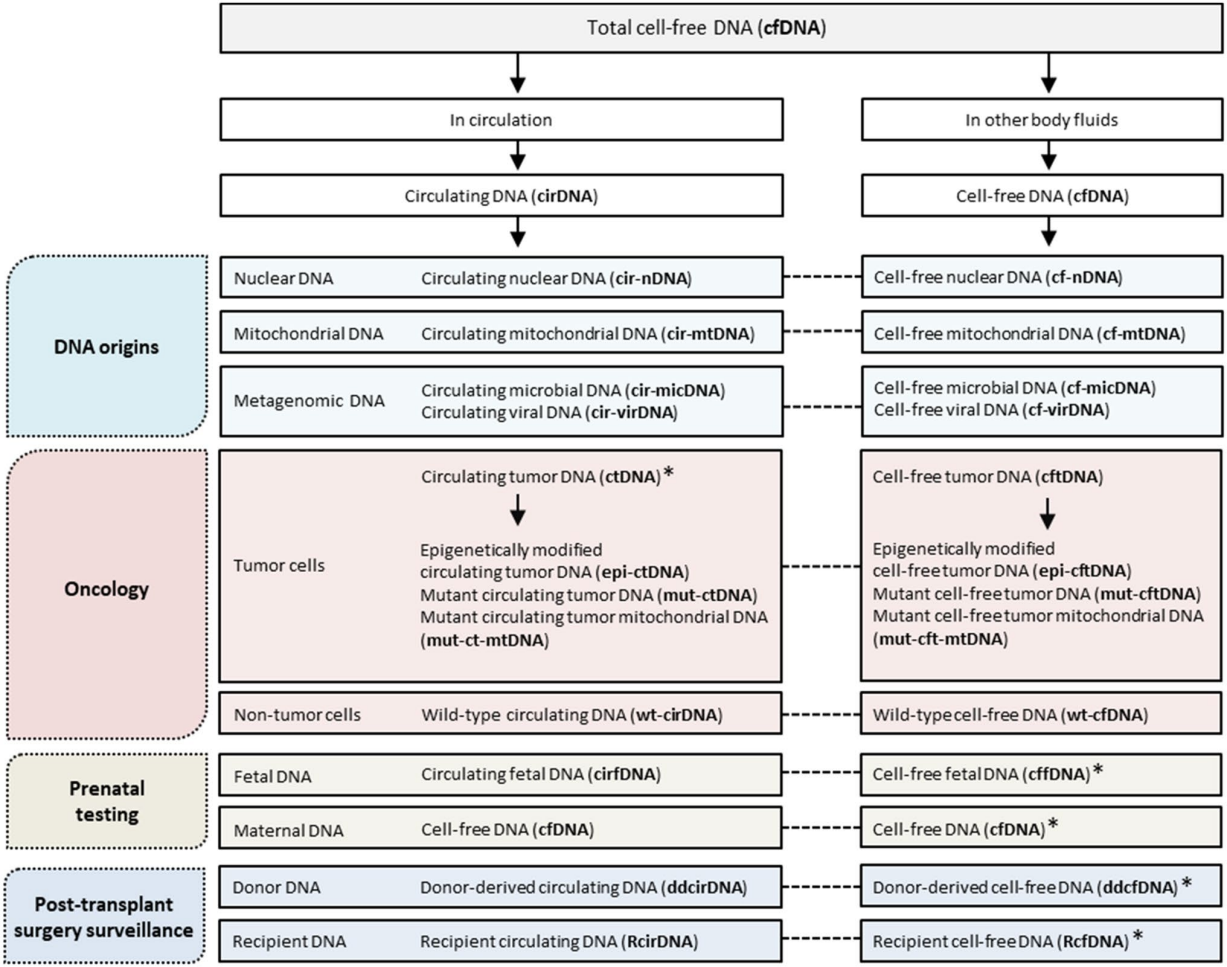
Nomenclature for cell-free DNA (cfDNA) in humans. The cfDNA content in biospecimens is biologically and structurally diverse. Assigning proper names to distinct types of cfDNA molecules thus represents an important step towards a common understanding of concepts among researchers. In this figure, we demonstrate how such a nomenclature can be devised in the fields of oncology, prenatal testing, and post-transplant surgery surveillance to differentiate between the different cfDNA types present in biospecimens collected from human body fluids. For the correct interpretation of this figure, three important points should be noted: First, the term cfDNA is appropriate for describing any free-floating DNA present in both circulating (i.e., blood and lymphatic fluids) and non-circulating body fluids (e.g., urine, saliva, and cerebrospinal fluid). Second, in cases where it is necessary (or when it would provide clarity) to distinguish between cfDNA coming from these different sources (e.g., parallel characterization of cfDNA in both body fluid types), the total cfDNA in non-circulating body fluids can retain the term cfDNA, while the total cfDNA in circulation can be termed circulating DNA (cirDNA). Similarly, for describing different cfDNA subtypes that are present only in circulation, the “cf” prefix in the respective abbreviations can simply be replaced by the prefix “cir”. Third, while the terms cirDNA and cfDNA can be used interchangeably, there exist common preferences for either type in certain cases, as indicated by the asterisk symbols. Therefore, the usage of the above terms is often appropriate only in specific experimental settings and other scenarios. Table 2 provides more information on this nomenclature, and indicates the different contexts in which each of the above terms may be useful

We hope that these recommendations will serve as a useful guideline for fellow researchers in the field or international associations such as the ICH (https://www.ich.org/) to systematically set up a universal cfDNA nomenclature in the near-future. Figure 1 summarizes the proposed nomenclature, whereas Table 2 indicates the context in which each term may be useful.

**Table 2:** Suggested usage of the proposed nomenclature

Category	Term	Abbreviations		Suggested to use when characterizing or referring to:
		Currently used	Newly proposed	
Familiar cfDNA types				
Total DNA	Extracellular DNA	exDNA		Any type of non-human DNA present in environmental compartments
	Cell-free DNA	cfDNA		Any type of DNA present in any human body fluids, mucosa, as well as cell culture supernatant
	Circulating DNA	cirDNA		Any type of DNA in circulation
Origins	Cell-free nuclear DNA		cf-nDNA	cfDNA of human nuclear DNA origin that needs to be distinguished from cfDNA of any other origin
	Cell-free mitochondrial DNA		cf-mtDNA	cfDNA of human mitochondrial origin that needs to be distinguished from cfDNA of any other origin
	Cell-free microbial DNA		cf-micDNA	cfDNA from any microbial origin
	Cell-free viral DNA		cf-virDNA	cfDNA from any viral origin
Oncology	Circulating tumor DNA	ctDNA		The aggregate circulating DNA population originating from a tumor
	Mutant circulating tumor DNA		mut-ctDNA	ctDNA with cancer-specific mutations
	Epigenetically modified circulating tumor DNA		epi-ctDNA	ctDNA with cancer-specific epigenetic modifications
	Mutant circulating tumor mitochondrial DNA		mut-ct-mtDNA	mut-ctDNA of mitochondrial origin that needs to be distinguished from other ctDNA types
	Wild-type circulating DNA		wt-cirDNA	Wild-type cirDNA originating from any cells
Prenatal testing	Cell-free fetal DNA	cffDNA		Fetal DNA in circulation
	Cell-free maternal DNA	cfDNA		Maternal DNA in circulation
Post-transplant surgery surveillance	Donor-derived cell-free DNA	ddcfDNA		Donor-derived cfDNA in organ transplant patients
	Recipient cell-free DNA		RcfDNA	Recipient-derived cfDNA in organ transplant patients
New/uncommon cfDNA types				
Various	Extracellular vesicle associated DNA		evDNA	cfDNA associated with extracellular vesicles
	Exosome associated DNA		exoDNA	cfDNA associated with exosomes
	Cell-free somatic DNA		cf-somDNA	cfDNA originating from somatic cells
	Cell-free germline DNA		cf-germDNA	cfDNA originating from germline cells
	Cell-fee nucleosomes		cfNucs	cfDNA or histone modifications associated with nucleosomes

When it is preferred or necessary to describe cfDNA types present in or isolated from circulating body fluids, the prefix “cf” in the above terms can be replaced with “cir”. Similarly, when it is preferred or necessary to describe cfDNA types present in or isolated from non-circulating body fluids, the prefix “cir” in the above terms can be replaced with “cf”

## Nomenclature for total cell-free DNA in different biological compartments

Whether in naked-form, protein-bound, or associated with extracellular vesicles (EVs), DNA that is liberated from the confinement of cells into any type of extracellular space is typically denoted by either of two general terms: extracellular DNA (exDNA) or cfDNA. While these two terms are often used interchangeably, clear biases in their use are apparent in the literature. The term exDNA is more frequently used to describe non-human DNA in the environmental context (e.g. soil, sediments, aquatic environments, and biofilm). One common exception here is the use of exDNA to describe both human and pathogen DNA captured in neutrophil extracellular traps (NETs) (Massberg et al. 2010), which are often referred to as exDNA traps. Conversely, the term cfDNA has been more widely adopted to describe various forms of DNA in the human context (i.e., body fluids). Therefore, it should be noted that the definitions of the terms exDNA and cfDNA, despite still often being used interchangeably, have diverged to the extent that they often mean different types of DNA. Accordingly, when cfDNA types only derived from human body fluids are under consideration, we give preference to the term cfDNA.

Two main types of human body fluids are commonly distinguished: those that are by definition circulating (blood and lymphatic fluids), and those that do not form part of the circulatory system (i.e., stool, saliva, sputum, urine, semen, amniotic fluid, cerebrospinal fluid, bile, bronchial lavage, esophageal brushings, breast discharge, tears, and cysts). Different approaches are used to assign nomenclature to cfDNA present in these different body fluids: (i) the total pool of cfDNA in both circulating and non-circulating body fluids can be described as cfDNA; (ii) when only cfDNA present in circulating body fluids is under consideration, either of the terms cfDNA or circulating DNA (cirDNA) can be used; (iii) in cases where it is necessary (or when it would provide clarity) to discriminate between cfDNA coming from these two different sources (e.g., when performing simultaneous characterization and comparison of cfDNA mutational profiles in plasma vs urine), the total cfDNA population in non-circulating body fluids can be defined as cfDNA, while the total cfDNA population in the circulatory system can be defined as cirDNA. However, these definitions overlap slightly which may also result in confusion and could be resolved by implementing separate nomenclature for DNA present in non-circulating vs. circulating body fluids, such as non-circulating cell-free DNA (nccfDNA) and circulating cell-free DNA (ccfDNA), respectively. An alternative approach may be to simply specify the body fluid in question, which in itself defines whether or not the fluid is circulating, e.g., plasma cfDNA, serum cfDNA, lymphatic fluid cfDNA, urine cfDNA, stool cfDNA, and cerebrospinal fluid cfDNA. While these are straightforward and viable approaches, it is unlikely to replace the popular terms cfDNA, cirDNA and ctDNA. One minor caveat, however, to be aware of when using the term cirDNA is its overlap with the nomenclature for circular DNA, an extrachromosomal DNA structure that can be present intracellularly and extracellularly (Kumar et al. 2017; Sin et al. 2020). In most cases, extrachromosomal circular DNA is abbreviated as eccDNA, but is in some cases also abbreviated as cirDNA.

Apart from describing cfDNA present in internal fluids, the term cfDNA can also be used to describe cfDNA recovered from any body fluids that have become separated from a body in a non-clinical setting, such as blood, sweat, and feces, which may represent valuable specimens for forensic casework (Quinones and Daniel 2012; Vandewoestyne et al. 2013). Similarly, the DNA present in human cell culture supernatant can be termed cfDNA (Bronkhorst et al. 2016).

## Nomenclature for cell-free DNA subtypes as defined by different origins

As discussed earlier, exDNA or cfDNA is found in different biological compartments in various organisms within the plant (Chayen and Norris 1953; Stroun et al. 1971, 1963) and animal kingdoms (Mandel 1948; Stroun et al. 1977, 1967). In humans, the origins of cfDNA can be highly heterogeneous due to the involvement of various tissues, organs, diverse mechanisms of release, endogenous microbes, and exogenous material. Thus, studies or reports combining data from different types of clinical samples, preclinical models, and even cell culture supernatants, should preferably distinguish the nature and origin of cfDNA by appropriate nomenclature. In such cases, as alluded to in the previous section, DNA extracted from non-circulating body fluids can be distinguished from DNA extracted from circulating body fluids by simply exchanging the “cf” prefix with “cir”. Pertaining to human cells, cfDNA of mitochondrial origin can be termed cell-free mitochondrial DNA (cf-mtDNA) (Chiu et al. 2003; Meddeb et al. 2019). In studies or reports where cfDNA of both mitochondrial and nuclear origin are concomitantly described, greater contrast between the two types may be facilitated by use of the term cell-free nuclear DNA (cf-nDNA). Pertaining to cfDNA of non-human origin, studies have demonstrated high levels of cfDNA fragments in human blood samples originating from known and unknown resident microorganisms (Burnham et al. 2016; Chiu and Alice 2019; Kowarsky et al. 2017; Stroun and Anker 1971). To describe the total cfDNA population originating from the metagenome of any microbe types that populate humans, the terms cell-free microbial DNA (cf-micDNA) or cell-free viral DNA (cf-virDNA) may be useful. In some cases, environmental nucleic acids, such as plant and bacterial DNA, can be taken up by humans through food, water, and other liquids and can be present in body fluids for extended time periods before it is degraded, excreted, or taken up by proximal cells (Spisák et al. 2013; Woegerbauer et al. 2020). Such cfDNA molecules could be collectively termed human metagenomic cfDNA.

On one hand, the vast number of cfDNA sources, the immense heterogeneity of cfDNA types, as well as the co-existence of genomes of various origins within human body fluids highlight the importance of caution when drawing conclusions on the measurement of these molecules. On the other hand, the high sensitivity and specificity of contemporary detection methods (e.g., Q-PCR, ddPCR and sequencing), integrated bioinformatics approaches, and wide range of genetic and epigenetic features that are open to interrogation enable increasingly enhanced characterization of cfDNA (Alborelli et al. 2019; Bronkhorst et al. 2019a; van der Pol and Mouliere 2019). This presents the unique opportunity of using cfDNA to reconstruct in silico the various genomes and epigenomes that are present in different regions of the human body in an unprecedented, minimally invasive manner. Harnessing this information may not only enable the identification of more disease-specific biomarkers, but, since cfDNA can be collected serially and over long periods, may also allow the monitoring of natural changes in the genome over time as well as allow the monitoring of dynamic genomic changes in response to a variety of environmental factors. This in turn may enable differentiation between a wide range of physiological states within individuals and between different individuals. As more scrutiny is placed on the various features of cfDNA and as increasingly more cfDNA studies on various physiological and pathological conditions are being conducted, the research field, and by extension the nomenclature, becomes more complex. In line with this, we will in the remainder of the paper focus on cfDNA nomenclature as it relates to three branches of medicine that have been positively impacted by cfDNA research.

## Cell-free DNA nomenclature in different branches of medicine

### Oncology

The term ctDNA is used to describe fragments of DNA that are released by a tumor into blood. While ctDNA is a well-accepted term, there are at least two potentially confounding ambiguities worth noting. First, in some cases ctDNA is used exclusively for denoting tumor-derived cirDNA fragments that harbor specific nuclear DNA mutations. In such cases, it is not clear whether the definition of ctDNA is also inclusive of DNA fragments that contain epigenetic modifications, as well as mitochondrial DNA. Second, in oncology the term cirDNA is often used to denote only wild-type cirDNA, but in most other cfDNA research fields cirDNA is a broad term used to describe the total population of plasma DNA, regardless of cellular origin.

In most cases, it is sufficient to define ctDNA simply as any type of cfDNA fragments that are released by a tumor and examined for the purposes of characterizing cancer. However, in certain cases (e.g., when ctDNA is used for the simultaneous analysis of genomic DNA mutations, epigenetic modifications, or mitochondrial DNA mutations in cancer patients) the above-mentioned ambiguities may become a source of confusion, which may be clarified through stratification of the different ctDNA subtypes. For this, the following nomenclature may be considered: (i) any cirDNA fragments that exhibit cancer-associated nuclear DNA mutations can be termed mutant circulating tumor DNA (mut-ctDNA); (ii) any cirDNA fragments that exhibit cancer-associated epigenetic modifications can be termed epigenetically modified circulating tumor DNA (epi-ctDNA); (iii) any circulating mitochondrial DNA (cir-mtDNA) fragments that exhibit cancer-associated mutations can be termed mutant circulating tumor mitochondrial DNA (mut-ct-mtDNA), and (iv) each of these three ctDNA types can be distinguished from any background DNA by the term wild-type cirDNA (wt-cirDNA). Although wild-type DNA is generally understood to mean DNA without any novel mutations, the term wt-cirDNA is co-opted here to denote any cirDNA molecules that do not contain the specific cancer-associated nuclear and mitochondrial DNA mutations or epigenetic modifications that are being interrogated, irrespective of their cellular origin. Similarly, mut-ctDNA, epi-ctDNA, and mut-ct-mtDNA are identified as such when they contain the cancer-specific markers that are absent in the wt-cirDNA as defined by the assays that are used, regardless of the specificity of the assay. In other words, whether the “variants” are truly cancer-defining or overlap with identical mutations or epigenetic modifications present in the cfDNA derived from normal tissues or other diseased tissues is not here taken into consideration. Indeed, this is one of the main issues that is currently being addressed by translational cfDNA research.

In line with this, a potentially confounding factor worth noting is the accumulating evidence for the presence of cancer-associated genomic alterations (e.g., mutations in the p53 tumor suppressor gene) in the clonal hematopoiesis (CH)-derived cfDNA of both cancer patients (Hu et al. 2018) and healthy individuals that do not have cancer and likely never will develop cancer (Anglesio et al. 2017; Fernandez-Cuesta et al. 2016; Genovese et al. 2014; Gormally et al. 2006; Newman et al. 2016). Experimental or in silico differentiation between CH-derived cfDNA and ctDNA molecules that bear the same mutations will provide a better understanding of the clinical implications of this phenomenon. This in turn will aid in the development of cfDNA assays with increased diagnostic sensitivity and specificity, thereby reducing the probability of misdiagnosing CH-derived cfDNA as malignancy. In most clinical settings, however, it is currently not possible to confidently trace cfDNA molecules back to their originating compartment and it is not yet clear how these molecules can be distinguished analytically. Therefore, there may not be an immediate need for terminology. However, an argument can be made that it may be useful to devise a provisional nomenclature based on the prospect that advances in our understanding of cfDNA biology would inform the development of methods that in the future allow more accurate differentiation between cfDNA molecules of different origins, notwithstanding similar DNA mutation profiles. This may, for example, become achievable through parallel characterization of cfDNA mutations and secondary features that exhibit tissue or cell-specific signatures, such as unique fragment sizes (Jiang et al. 2015; Marass et al. 2020; Mouliere et al. 2018; Sanchez et al. 2018), endpoint motifs (Cristiano et al. 2019; Jiang et al. 2020), nucleosome density/positioning (Snyder et al. 2016; Ulz et al. 2019b, 2016), or methylation patterns (Lehmann-Werman et al. 2016; Moss et al. 2018; Zemmour et al. 2018). Such a terminology may look as follows: DNA released from any non-tumor cells are termed non-tumor circulating DNA (NT-cirDNA). NT-cirDNAs that do not exhibit cancer-associated mutations can be termed wild-type non-tumor circulating DNA (wt-NT-cirDNA). Conversely, NT-cirDNAs that exhibit cancer-associated mutations can be termed mut-NT-cirDNA. Thus, in addition to cirDNA of microbial, viral and exogenous origin, the total cirDNA profile obtained following isolation of DNA from a cancer patient’s blood sample can be comprised of both ctDNA (mut-ctDNA, epi-ctDNA, and mut-ct-mtDNA) and NT-cirDNA (wt-NT-cirDNA and mut-NT-cirDNA). Note, the term wild type is often incorrectly used by oncologist as meaning wild type at the position/locus of specifically screened mutations.

**Fig. 2:**
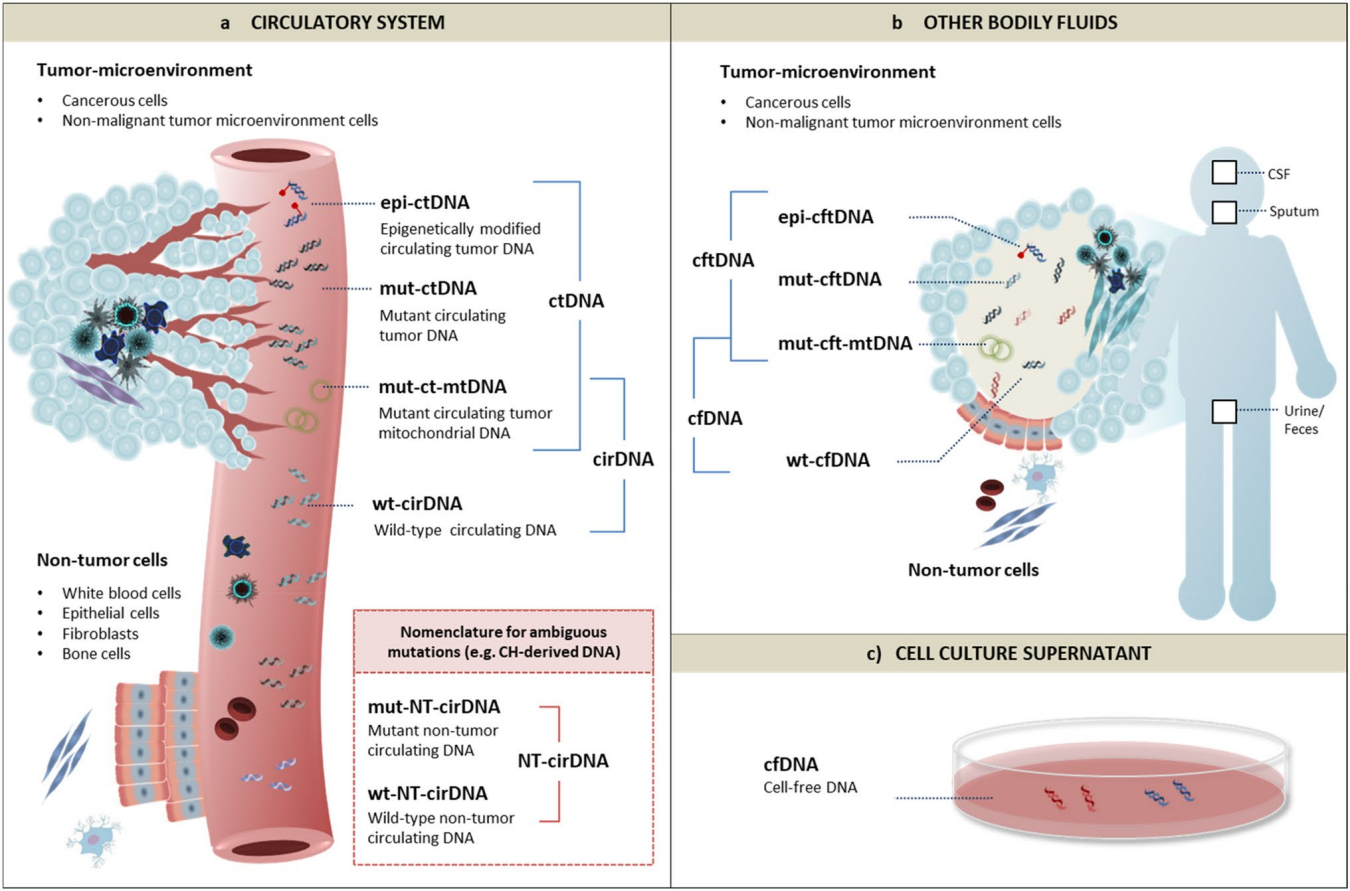
Nomenclature for cell-free DNA (cfDNA) in oncology. In the above representation, we demonstrate how nomenclature can be devised to differentiate between the different types of cancer cell-derived cfDNA fractions present in samples collected from **a** the circulatory system (blood and lymphatic fluids), **b** other bodily fluids (e.g., urine, feces, cerebrospinal fluid, bronchial lavage, and sputum), and **c** cell culture supernatant. Table 2 summarizes the different contexts in which each of the above terms may be useful

Since most cancer studies have thus far been based on the characterization of DNA extracted from circulation, the focus in this section was placed on the nomenclature of DNA in circulation. However, it is becoming clearer that some non-circulating body fluids contain a richer source of specific tumor-derived cfDNA (reviewed in (Bronkhorst et al. 2019b)). Thus, when considering DNA extracted from body fluids other than blood or serum, the prefix “cir” can be replaced by “cf” (Table 2). Nomenclature for cfDNA in cancer is summarized in Fig. 2.

### Prenatal testing

**Fig. 3:**
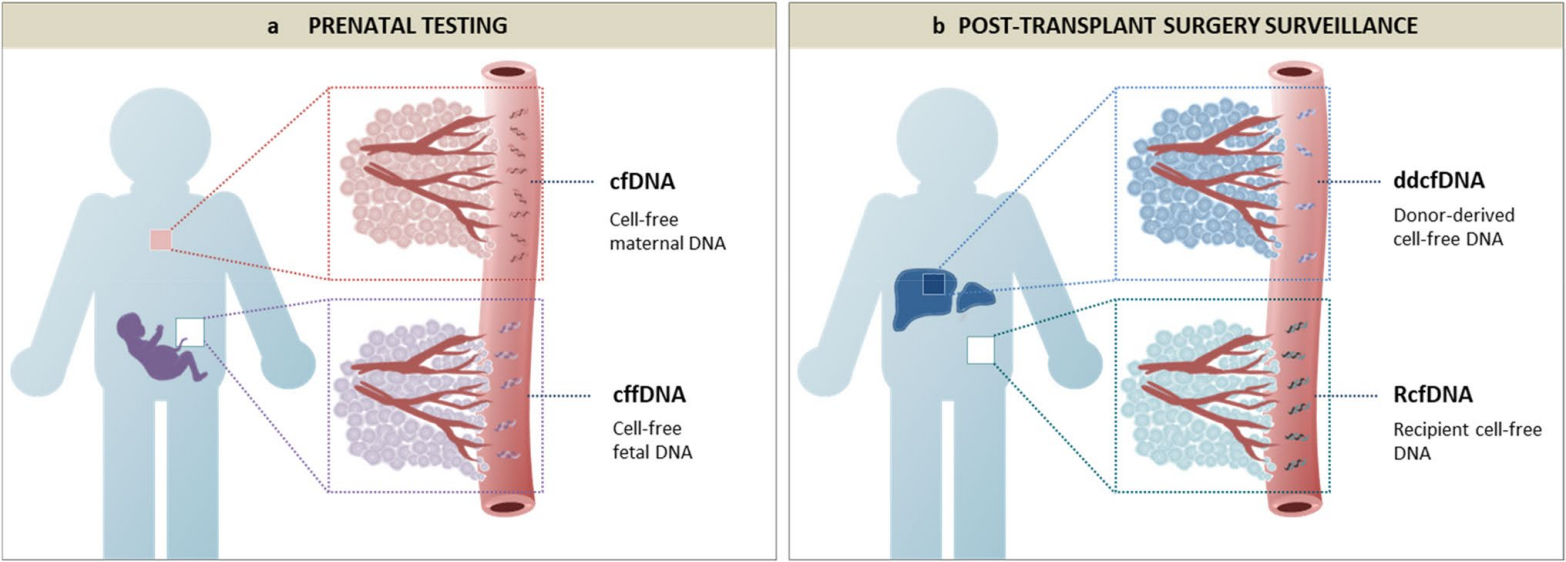
Nomenclature for cell-free DNA (cfDNA) in **a** prenatal testing and **b** post-transplant surgery surveillance. Table 2 summarizes the different contexts in which each of the above terms may be useful

Molecular analysis of plasma and serum DNA during human pregnancy has led to the discovery that maternal blood contains cfDNA of both fetal and maternal origin (Amicucci et al. 2000; Lo et al. 1997). The origin of the cell-free fetal DNA (cffDNA) is the cytotrophoblast; so strictly speaking, it is placental DNA rather than fetal DNA that circulates in maternal blood (Alberry et al. 2007; Flori et al. 2004). However, the term “cffDNA” has gained universal acceptance in the prenatal literature and no change to this practice is proposed here. This uniquely accessible source of fetal DNA has opened up new possibilities for prenatal diagnosis and screening (Lo et al. 1998). The use of cffDNA for fetal molecular diagnostics is called noninvasive prenatal diagnosis (NIPD) and has clinical applications in the determination of fetal blood group, fetal sex, and an expanding range of monogenic conditions (Chitty and Lo 2015). This should be distinguished from the use of cffDNA for the detection of fetal chromosome conditions such as trisomy 21, which has been variously called noninvasive prenatal testing (NIPT), noninvasive prenatal screening (NIPS) and maternal cfDNA screening (Benn et al. 2013; Gregg et al. 2016; Taylor-Phillips et al. 2016). The use of cffDNA for aneuploidy detection is a screening test, and diagnostic confirmation with invasive testing (chorionic villus sampling or amniocentesis) is still required after a high-risk result. The phenomenon of fetoplacental mosaicism is the biological reason why NIPT cannot achieve diagnostic accuracy for chromosome conditions, as the placental karyotype (which characterizes the plasma cfDNA profile) is not always representative of the true fetal karyotype (Brison et al. 2018; Pertile et al. 2017). CffDNA describes the fetoplacental-derived fraction of the maternal plasma cfDNA (the total DNA deriving from both the fetus and the mother should simply be termed as cfDNA). The ‘fetal fraction’ (FF) is the proportion of total maternal plasma cfDNA that arises from the fetoplacental unit, that is FF = cffDNA/(cffDNA + maternal plasma cfDNA). The FF is an important quality control metric in NIPT and is routinely calculated by most laboratories during the clinical workflow (Hui and Bianchi 2020). Nomenclature for cfDNA in prenatal testing is summarized in Fig. 3a.

### Post-transplant surgery surveillance

Characterization of donor cfDNA derived from a transplanted organ is emerging as a potentially useful tool to monitor post-transplant allograft rejection, dysfunction, and injury (Bloom et al. 2017; De Vlaminck et al. 2014; Moss et al. 2018; Schütz et al. 2017; Sharon et al. 2017; Sigdel et al. 2019). A method called “genome transplant dynamics” (GTD) is one of the first approaches developed for discriminating between donor and recipient cfDNA molecules, and is based on the quantification of unique single-nucleotide polymorphisms distributed across the genome (Snyder et al. 2011). To-date, several terms have been used to describe donor cfDNA, including donor-specific cfDNA (cfdDNA), donor-derived cfDNA (either dcfDNA, ddcfDNA, or dd-cfDNA), cell-free donor-derived DNA (cfdDNA), and graft-derived cell-free DNA (GcfDNA). To simplify this, we suggest using only the term donor-derived cfDNA (ddcfDNA) for describing the cfDNA originating from the donor organ and to distinguish ddcfDNA from the host cfDNA using the term recipient cfDNA (RcfDNA) (nomenclature summarized in Fig. 3b).

## Expansion of cell-free DNA nomenclature in the future

**T**he landscape of cfDNA research will continue to change, likely prompting further diversification and expansion of nomenclature. There are at least three major drivers of this change:

First, there is a rapidly growing interest in the research field. Beyond the domains of oncology, prenatal testing and post-transplant surgery surveillance, cfDNA has been identified as a potential biomarker in various other conditions, such as cardiovascular disease (Polina et al. 2020), autoimmune disease (Duvvuri and Lood 2019), sepsis (Ullrich et al. 2020), trauma (Gögenur et al. 2017), aging (Teo et al. 2019), physical exhaustion (Breitbach et al. 2012), and may even become a useful clinical tool in veterinary science (Goggs et al. 2020).

Second, an increasing number of studies are aimed towards improving our understanding of how to best harness the diverse biological and pathological information encoded in cfDNA for the management of various diseases. For example, information on the level of cfDNA tissue-of-origin may be vital for understanding and treating diseases that are typically very difficult to examine and monitor noninvasively, such as neurodegenerative, inflammatory, and ischemic diseases. A recent study has shown that tissue-specific methylation patterns are conserved in cfDNA fragments and can be used to identify cell-death in heart tissues (Zemmour et al. 2018). In addition, sophisticated approaches using a reference methylation atlas recently enabled the differentiation of cfDNA fragments isolated from healthy human plasma based on the relative contribution of different cell types, such as white blood cells (55%), erythrocyte progenitors (30%), vascular endothelial cells (10%) and hepatocytes (1%) (Moss et al. 2018).

Third, several landmark studies have recently demonstrated the importance of an improved understanding of both the physico-chemical properties of cfDNA, as well as the biological and physiological factors that modulate these properties. This knowledge will not only aid in the identification of more disease-specific cfDNA features, but also inform the development of strategies that maximize the chances of detecting target cfDNA molecules, thereby increasing the diagnostic sensitivity and specificity of clinical assays, such as; the selection of patient conditions that either favor the release of target molecules or limit the release of background molecules into the body fluids in question prior to sampling; optimization of preanalytical procedures that preserve target molecules and limit the incidence of contaminating DNA; tailoring or development of extraction procedures that are either biased towards the capture of specific cfDNA molecules or the elimination of non-specific DNA molecules. Therefore, in keeping with these recent important findings, it may in the near-future become necessary to devise nomenclature for distinguishing between (i) cytoplasmic vs. cell-surface bound cfDNA (Tamkovich and Laktionov 2019), (ii) cfDNA fragments that possess different epigenetic signatures (e.g., unique DNA fragmentation patterns and endpoint motifs, methylation patterns, nucleosome positioning and transcription factor binding sites) (Sanchez et al. 2018; Snyder et al. 2016; Sun et al. 2018; Ulz et al. 2019a), (iii) cfDNA fragments that exhibit different sizes, (iv) cfDNA fragments that originate from somatic cells vs. germline cells, which may be termed cell-free somatic DNA (cf-somDNA) and cell-free germline DNA (cf-germDNA), respectively, (v) cfDNA complexed or associated with different proteins and other subcellular components—for example, studies have shown significant portions of cfDNA to be associated with (a) histone proteins in nucleosomal structures, which may be termed cell-free nucleosomes (cfNucs), (b) extracellular vesicles, which may be termed extracellular vesicle associated DNA (evDNA), (c) specific extracellular vesicles such as exosomes, which may be termed exosome associated DNA (exoDNA), (d) small lipoprotein complexes, (e) fragments of cellular membranes, and (f) neutrophil extracellular traps (NETs) released from polymorphonuclear neutrophils, which are structures composed of DNA, histones, granules and enzymes (Aucamp et al. 2018; Thierry et al. 2016).

Lastly, in this report, we focused on the terminology of cfDNA. However, in recent years, various types of cell-free RNA molecules have emerged as potentially powerful biomarkers (e.g., non-coding RNAs such as miRNAs, lncRNAs, and rRNAs). It is feasible that much of the nomenclature proposed in this paper can be transposed to the cell-free RNA field by simply exchanging “DNA” with “RNA” where appropriate. Moreover, when both DNA and RNA are under consideration in the same instance the abbreviation prefixes can be followed by “NAs” to denoted nucleic acids, instead of “DNA” or “RNA”. In this regard, it is worth noting that a specific type of DNA and RNA is circular in structure and are sometimes termed cirDNA and cirRNA, respectively. To avoid confusing these terms with circulating DNA (also abbreviated as cirDNA) and circulating RNA (also abbreviated cirRNA), we suggest using the more common nomenclature when it is necessary to refer to circular RNA (circRNA) or circular DNA, including extrachromosomal circular DNA (eccDNA) or covalently closed circular DNA (cccDNA).
